# Supplementary material for: Climate Adaptation Strategies for Maintaining Rice Grain Quality in Temperate Regions
Source: Biology (Basel). 2025 Jul 2;14(7):801. doi: 10.3390/biology14070801 (PMC12292368; doi:10.3390/biology14070801)
Supplement: Supplementary file 1 [file biology-14-00801-s001.zip › biology-3696707-supplementary.html]

Rice Stress-Quality Integration Network


# 🌾 Rice Stress-Quality Integration Network

Evidence-based molecular pathways controlling temperate rice grain quality under climate stress

#### Supplementary Figure 1. Rice Stress-Quality Integration Network Model

**Network Architecture:** This interactive model demonstrates validated molecular networks controlling rice grain quality under climate stress. The multi-hub architecture comprises OsSnRK1A Hub as the central energy-stress regulator (regulating specific quality-related genes including OsNADH-GOGAT2 and defense genes) that coordinates with OsONAC Hub (NAC transcription factor family with stress-responsive expression), OsTOR Hub (growth-defense integration hub operating antagonistically to OsSnRK1A), and OsSnRK2 Hub (10-member kinase family with OsSAPK8 as central ABA signaling component).

**Environmental Sensors:** Four specialized sensor systems detect stress conditions: ROS Sensors (9 NADPH oxidases OsRbohA-I generating reactive oxygen signals), Ion Sensors (HKT transporter family critical for salinity tolerance and ion homeostasis), Hormone Sensors (ABA biosynthesis and perception system including OsNCED1-5 and OsPYL/RCAR1-13 receptors), and Ca2+ Sensors (calcium-dependent signaling including OsCPK12 and OsCaM1-1). These sensors activate regulatory hubs through documented molecular interactions, triggering coordinated pathway responses.

**Quality Pathway Integration:** Four major biochemical pathways (shown in pink) coordinate grain quality formation: Starch (biosynthesis enzymes including OsAGPL1/3, OsGBSSI-Wx gene, SSS/SBE families), Protein (nitrogen metabolism via glutamine synthetase family), Aroma (BADH2-mediated 2-acetyl-1-pyrroline synthesis), and Antioxidant (ROS scavenging systems including SOD, CAT, APX families). Each pathway influences specific quality traits: amylose content (AC), protein content (PC), aroma compound concentration (2-AP), chalkiness (CHK), and milling quality (MQ).

**Stress Response Patterns:** Model parameters reflect experimental evidence showing differential responses to environmental stresses: heat stress disrupts key enzymes (particularly OsGBSSI), leading to amylose reduction and increased chalkiness; drought stress enhances protein accumulation through ABA-mediated responses and altered C:N partitioning; salt stress activates ion homeostasis mechanisms with moderate quality impact; and cold stress reduces enzyme activity affecting grain filling. Interactive controls allow exploration of these stress patterns and pathway interactions.

**Copyright:** Interactive network visualization licensed under CC BY 4.0. Copyright © 2025 Fernando, Ovenden, Sreenivasulu, and Butardo.

**Note:** This visualization represents a synthesis of current rice-specific literature. The core pathways and their connections to grain quality traits are well-documented, but the precise regulatory mechanisms and quantitative stress responses may vary by genotype and environmental conditions. The model is intended as an educational overview of the integrated stress-quality network and should be interpreted accordingly.

### Environmental Stress

**Baseline**  
Optimal growing conditions

**Drought**  
Water deficit (-0.5 to -2.0 MPa)

**Salinity**  
NaCl stress (50-100 mM)

**Heat**  
Temperature >32°C

**Cold**  
Temperature <17°C

### Stress Intensity

Mild
Moderate
Severe
Extreme

### Network Response

#### Baseline Conditions

Normal rice grain development under optimal environmental conditions.

1.0x

Average Hub Activity

0%

Quality Change

#### Network Components

OsSnRK1A Hub (Master)

OsONAC Hub (NAC TFs)

OsTOR Hub

OsSnRK2 Hub

Environmental Sensors

Biochemical Pathways

Quality Traits (AC, PC, 2-AP, CHK, MQ)
